# Polyimide/SU-8 catheter-tip MEMS gauge pressure sensor

**DOI:** 10.1007/s10544-012-9661-8

**Published:** 2012-05-26

**Authors:** Willyan Hasenkamp, David Forchelet, Kristopher Pataky, Jimmy Villard, Harald Van Lintel, Arnaud Bertsch, Qing Wang, Philippe Renaud

**Affiliations:** 1 École Polytechnique Fédérale de Lausanne, Lausanne, Switzerland; 2 Microsystems Laboratory, STI-LMIS4, Station 17, EPFL, Lausanne, Switzerland; 3 Microsystems Laboratory, STI-LMIS1, Station 17, EPFL, Lausanne, Switzerland; 4 Klinikum rechts der Isar, Technische Universität München - TUM, Munich, Germany; 5 Department of Medicine, Division of Nephrology and Hypertension, CHUV, Lausanne, Switzerland

**Keywords:** Pressure sensor, Pressure monitoring, Biosensor, Strain sensor, Catheter-tip, Polyimide, SU-8

## Abstract

This paper describes the development of a polyimide/SU-8 catheter-tip MEMS gauge pressure sensor. Finite element analysis was used to investigate critical parameters, impacting on the device design and sensing characteristics. The sensing element of the device was fabricated by polyimide-based micromachining on a flexible membrane, using embedded thin-film metallic wires as piezoresistive elements. A chamber containing this flexible membrane was sealed using an adapted SU-8 bonding technique. The device was evaluated experimentally and its overall performance compared with a commercial silicon-based pressure sensor. Furthermore, the device use was demonstrated by measuring blood pressure and heart rate *in vivo*.

## Introduction

Polymer-based microelectromechanical systems (MEMS) are increasingly being used in biomedical applications (Grayson et al. 2004). Inexpensive well-established micromachining techniques, material versatility, robustness and biocompatibility constitute some of its potential advantages over conventional biomedical device fabrication methods (Liu 2007). A number of polymer-based devices has been realized including implantable microelectrodes (Metz et al. 2004), microsystems for neural prostheses (Mercanzini et al. 2008), microfluidic devices (Metz et al. 2001), temperature sensors (Moser and Gijs 2007), humidity sensors (Zeng et al. 2006), sensors for orthopedic implants (Arami et al. 2011), tactile sensors (Fan et al. 2004) and pressure sensors (Mannsfeld et al. 2010; Hill et al. 2007; Lim et al. 2005; Leonardi et al. 2003).

Polyimide has been used in various aspects of microelectronics fabrication including multilevel interconnect technology and flexible electronics (Frazier 1994). Polyimide is a highly suited material for micromachined medical devices, as it is biocompatible, electrically insulating, thermally stable and exhibits low water uptake (Richardson et al. 1993). Additionally, polyimide has a high magnetic permeability, offers a low residual structural stress, and suits for fabrication of dynamic microsystems since it has proven to be a durable material in MEMS devices (Frazier 1995).

SU-8 is a negative epoxy-based photoresist which has been widely used for packaging MEMS (Zine-El-Abidine and Okoniewski 2009) and for creating high aspect ratio features using conventional photolithography (Dellmann et al. 1998). SU-8 micromachining provides a short device manufacturing cycle. It can be bonded and laminated, reducing the fabrication cost and complexity when compared with conventional silicon technology (Hill et al. 2007). SU-8 can be processed at low temperatures and cures into a glassy biocompatible element with reduced biofouling (Grayson et al. 2004).

In general, equipment requirements for polyimide/SU-8 MEMS processing are similar to those of conventional microfabrication techniques, hence commonly available cleanroom equipment and materials can be utilized for processing without extensive development. We have chosen to integrate polyimide and SU-8 into the fabrication of a catheter-tip MEMS gauge pressure sensor. Polymer-based pressure sensors have many *in vivo* applications such as intracranial pressure monitoring in case of head trauma (Li et al. 2010), intraocular pressure evaluation for glaucoma (Leonardi et al. 2004), blood pressure and heart rate monitoring for cardiovascular assessment (Grayson et al. 2004). In biomedical research, genetically modified mice are met with growing interest, since diseases can be expressed and studied in a convenient platform, creating needs for more compact and precise devices. Miniaturized pressure sensors offer potential improvement over fluid-filled catheters (Glantz and Tyberg 1979), as they can be mounted on the catheter-tip directly which avoids signal damping due to pressure dynamics. In fluid-filled systems, the catheter is inserted into the target site and transmits the pressure to a remote sensor through the fluid column—effectively creating a low-pass filter impairing the monitoring of rapid fluctuations (Wang et al. 2004). Furthermore, miniaturize sensors can decrease the risk of thrombosis, embolism and infections since common available commercial sensors are oversized and will not fit into specific small vessels or cavities (Hill et al. 2007).

This paper presents the development of a 400 μm-wide hybrid polymer-based (Polyimide/SU-8) strain gauge pressure sensor mounted at the tip of a Pebax catheter (inner diameter/outer diameter: 0.20/0.48 mm). Finite Element Analysis (FEA) was used to investigate the distribution of the stress and strain on the polyimide membrane and to evaluate the device dimensions and the appropriate locations for the placement of the sensing elements. The sensing elements consist of piezoresistive thin-film metallic wires embedded in a flexible polyimide membrane. A sealed pressure chamber is created upon the polyimide membrane using an adapted bonding technique, commonly employed to fabricate microchannels (Metz et al. 2001). Next, the device is evaluated experimentally. First, its performance is compared with a commercial silicon-based pressure sensor. Next, the response of both sensors are compared when exposed to high-speed pressure fluctuations. Finally, the device is used *in vivo* to measure blood pressure of a mouse.

## Finite element analysis

**Fig. 1:**
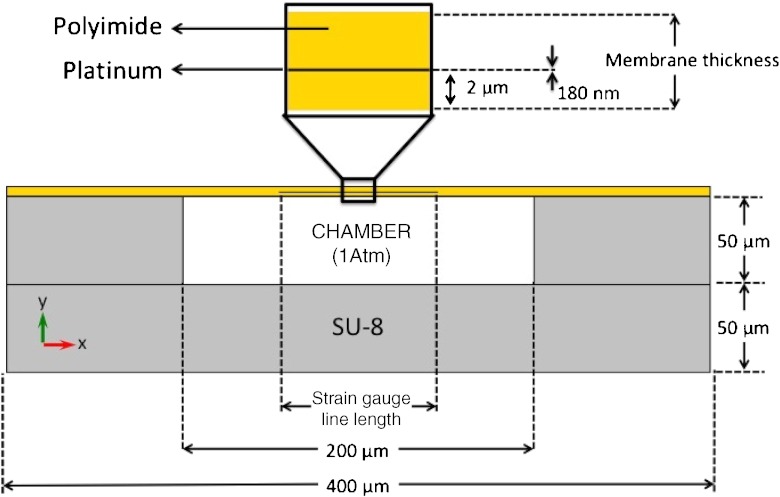
CAD model comprising the polyimide membrane, the platinum as piezoresistive material and the SU-8 enclosed chamber (cross-section along the device width)

The chosen method for measuring the pressure is to detect mechanical deformations in a thin polyimide membrane supported by a sealed pressure chamber made in SU-8. Finite Element Analysis (FEA) is an effective tool to investigate the distribution of the stress and strain in various types of structures (Kasi et al. 2011). In this article FEA was used to investigate the stress and strain in the polyimide (PI) membrane in order to determine the appropriate membrane thickness, to assure no plastic deformation occurs during the operation of the device and to assist with the placement of the strain gauges within the device. A 2D Computer Assisted Design (CAD) model was built into a commercial FEA software, COMSOL Multiphysics (v4.2a). The 2D CAD model is presented in Fig. 1, and comprises a PI membrane, platinum wires as the piezoresistive element and a pressure chamber made of SU-8. The total width of the device was limited to 400 μm in order to match the tip-width of a Pebax catheter (inner diameter/outer diameter: 0.20/0.48 mm). The 100 μm-wide, 50 μm-thick SU-8 pressure chamber was designed to prevent deformations other than those of the PI membrane. Other geometrical dimensions (i.e. membrane thickness and strain gauge line length) were chosen from the results of the simulation.

The FEA was modeled in the Structural Mechanics module of Comsol in stationary mode assuming a linear elastic behavior for all parts. The fundamental considerations in this approach are to assume a small strain (or stress) and a linear relationship between the components stress/strain (i.e. Young’s modulus). Moreover, the results are valid only for stress states that do not produce plastic deformation, therefore stresses above the yield point (YP) of the materials will be discredited. The material properties necessary to perform the FEA are: density (*ρ*), Young’s modulus (*E*) and Poisson’s ratio (*ν*). The assumed values are presented in Table 1.

**Table 1:** Material properties used in the FEA

Material	*ρ* (kg/m^3^)	*E* (*Pa*)	*ν*
Polyimide	1,400	8 × 10^9^	0.2
Platinum	21,450	145 × 10^9^	0.38
SU-8	1,200	4.4 × 10^9^	0.22

The following constraints were added to the model. A boundary condition load was applied around the device to account for pressure changes outside the device. Another boundary condition load was defined inside the device, corresponding to the inner part of the SU-8 pressure chamber. This constraint accounts for pressure changes inside the device due to changes in the cross-sectional area of the pressure chamber induced by the external change in pressure. Equation 1 defines the pressure change as a function of the cross-sectional area, where *Δp* corresponds to the pressure change inside the device, *p*_0_ is the gauge pressure inside the pressure chamber (considered to be the ambient pressure in which the the device was fabricated), *A*_0_ is the pressure chamber cross-sectional area at ambient pressure (10$^{5}\,\upmu$m^2^), *A* is the pressure chamber cross-sectional area induced by the external changes in pressure and *γ* is the adiabatic index (1.4 for air). To complete the model, a fixed constraint is defined at the bottom of the device to avoid overall device displacement which can introduce discrepancies in the simulation.

1 $$ \Delta p = p_{0} \left ( \left (\frac{A_{0}}{A} \right )^{\gamma}-1 \right ) $$

An unstructured progressive triangular meshing algorithm was utilized for meshing the model with minimum and maximum element size of 8 nm and 4 μm, respectively. The total number of elements generated by the meshing was 34,088. The convergence criteria was establish by a MUMPS solver.

## Results of the finite element analysis

**Fig. 2:**
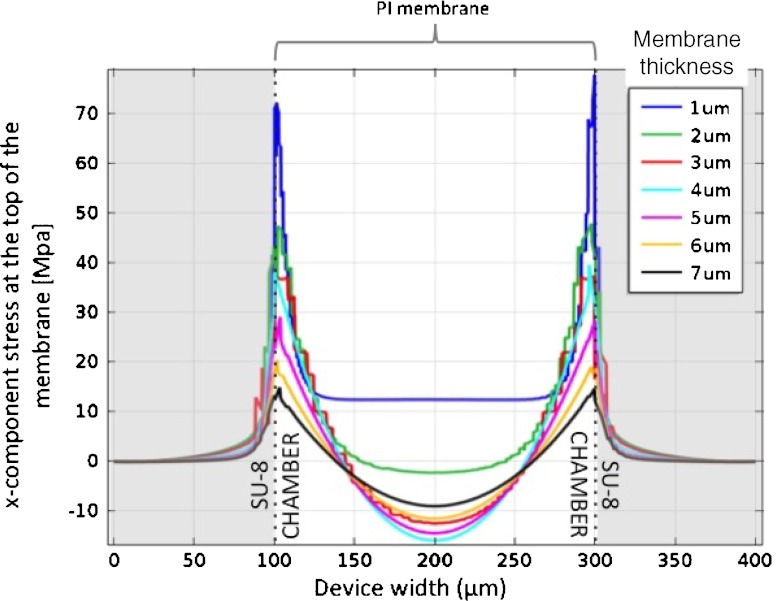
x-component of the membrane stress along the device width as a function of the membrane thickness, in the transverse plane situated at the *top* of the PI membrane

FEA was first used to evaluate the acceptable thickness values for a PI membrane closing the SU-8 chamber. The PI has a yield point (YP) of approximately 65 MPa and larger values of stress result in plastic deformation of PI, permanently damaging the device. The simulation was performed in a simplified model using a monolitic PI membrane without platinum tracks and exposed to a pressure of 350 mmHg, corresponding to more the twice the blood pressure for mice. Figure 2 shows the evolution of the x-component of the membrane stress with the membrane thickness, along the device width and in the location where the membrane stress is maximum (in the transverse plane situated at the top of the PI membrane). Clearly visible is the presence of high values of stress where the PI membrane is attached to the SU-8 chamber, and the limit of plastic deformation of PI is reached at those locations for membranes thinner than 3 μm. For the fabrication of the device, PI membranes were therefore chosen to be 5 μm in total thickness.

**Fig. 3:**
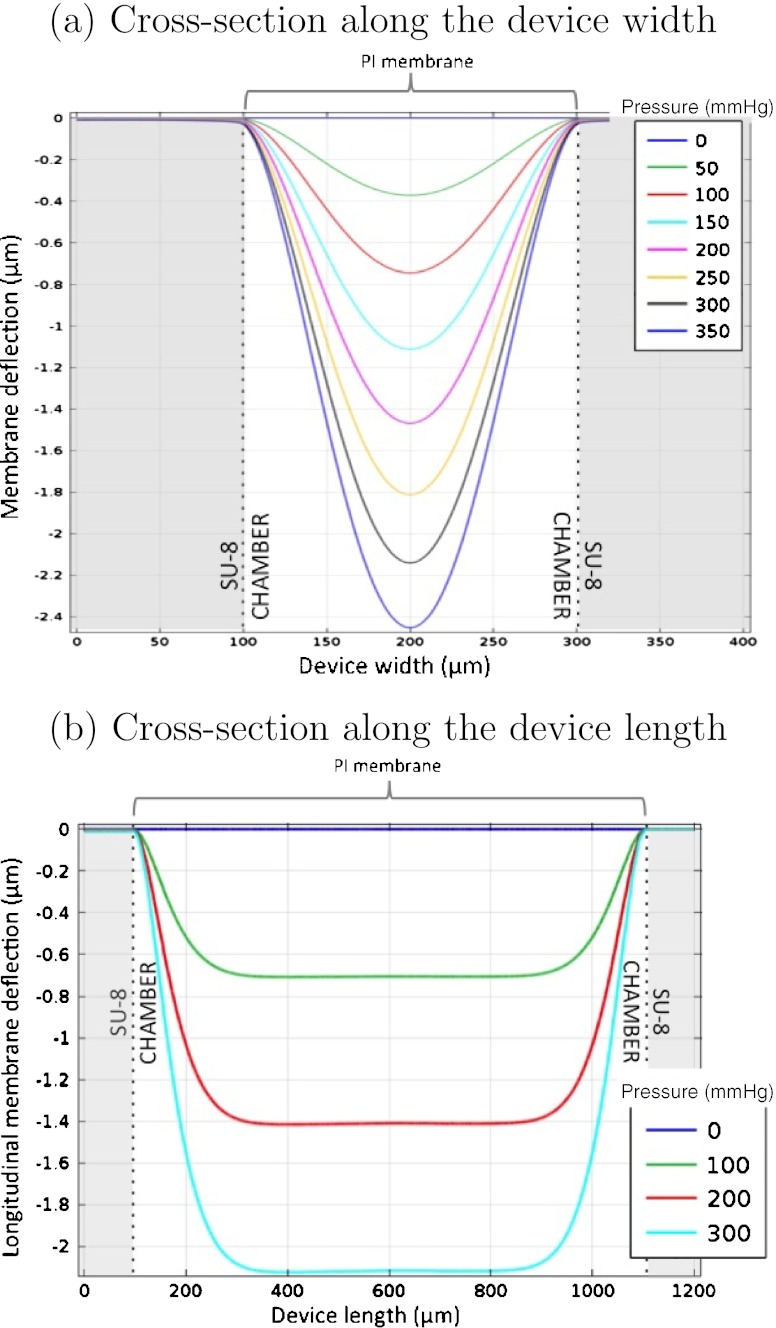
PI membrane deflection with the applied pressure, in the transverse plane situated at the *top* of the PI membrane. (**a**) Width cross-section and (**b**) Length cross-section

The PI membrane deflection was also analyzed, both in width and in length, for different values of pressures. These pressure variations were added to the atmospheric pressure of 1 atm. Figure 3 presents the results for a 5 μm thick PI membrane deflection in response to applied pressure, in the transverse plane situated at the top of the PI membrane. Figure 3(a) shows the deflection in a cross-section along the width of the device and Fig. 3(b) presents the deflection in a cross-section along the length of the device. Both graphs in Fig. 3 show no deflection of the PI membrane at 0 mmHg (corresponding to the atmospheric pressure). This is expected since the pressure inside the sensor’s pressure chamber was set to 1 atm (matching the packaging ambient pressure). At the pressure of 300 mmHg the computed deflection of the membrane is approximately 2.1 μm with the two graphs in Fig. 3 showing a linear deformation of the membrane with the applied pressure. In Fig. 3(b) we verify a constant strain region in the center of the membrane along approximately 0.6 mm in length. Therefore, we chose to fabricate strain gauges having a maximum width of 600 μm to fit into a 1 mm long membrane.

**Fig. 4:**
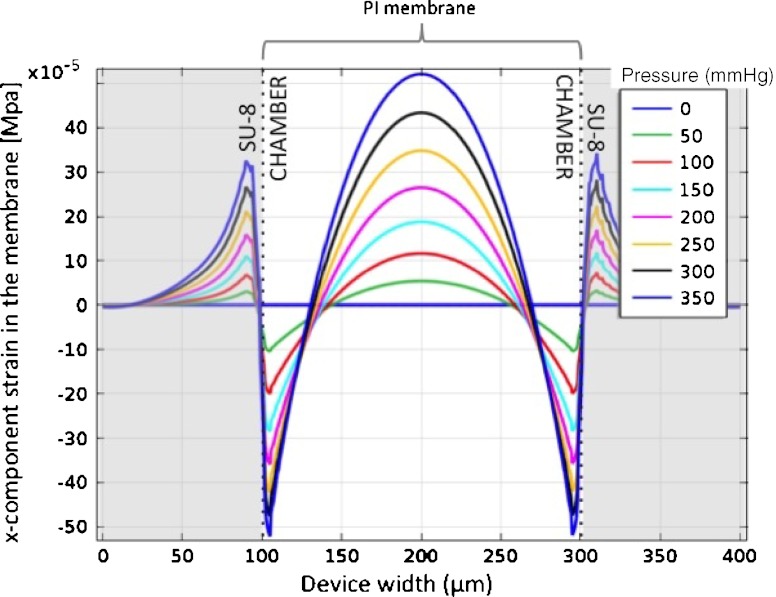
x-component of the membrane strain with the applied pressure, in the transverse plane situated at the *top* of the PI membrane and along the device width. 2D model without platinum trace

To define the line length of the active strain gauge a FEA simulation was performed to investigate the strain on the PI membrane. Figure 4 shows the x-component of the strain of a 5 μm thick membrane as a function of the applied pressure, in the transverse plane situated at the top of the PI membrane, along the device width. Highly strained regions are present in the middle of the device (mostly compressive) and close to the SU-8 walls (both compressive and tensile), however the central part of the membrane sustains the strain over a longer region, without changing from compressive to tensile strain. Therefore, the active strain gauge of the device was placed in the central part of the membrane. To avoid tensile strain and keep a safe margin for alignment during fabrication, a line length of 90 μm was chosen for the active strain gauge. To fit the defined maximum strain gauge area width of 600 μm and simplify the photolithography process during device fabrication the line width of the strain gauges was defined to be 4 μm with line spacing of 6 μm.

**Fig. 5:**
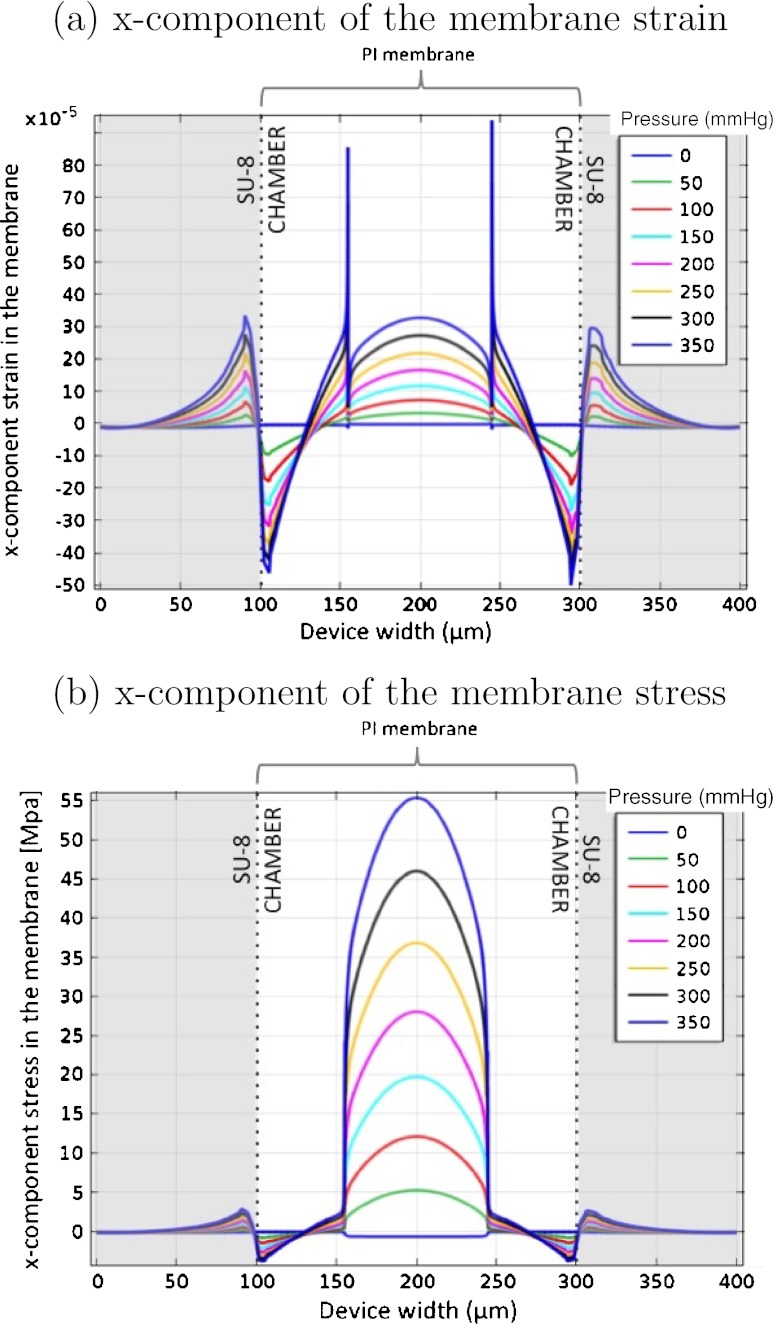
x-component of the membrane (**a**) strain and (**b**) stress with the applied pressure, in the transverse plane situated at 2 μm from the *bottom* of the PI membrane and along the device width. 2D model including the defined platinum line embedded into the polyimide membrane

Having defined the location and dimensions of the active strain gauge to be placed as sensing element in the PI membrane, a second FEA modeling step was performed using a model in which the metal tracks are considered in order to evaluate their effect on the PI membrane strain and stress. The platinum metal tracks were inserted 2 μm from the bottom of the PI membrane. Figure 5 presents the impact of the 90 μm line length strain gauge on the x-component of the strain (Fig. 5(a)) and stress (Fig. 5(b)) in the PI membrane with the applied pressure, in the transverse plane situated at 2 μm from the bottom of the PI membrane and along the device width. The highly strained regions seen close to the metal line (at 160 μm and 240 μm in width) are artifacts due to the interactive convergence and discretization methods governing the algebraic expressions in a discrete domain (e.g. finite-element). The stiffening effect of the piezoresistive material embedded in the PI membrane is clearly visible in the central part of the graph. When compared to the results from Fig. 4 a 32% reduction of the strain was found as a consequence of the presence of the metal tracks in the PI membrane. In Fig. 5(b) the stress on the platinum domain is much higher than in the PI material, however the YP of platinum is ~1 GPa. Therefore, no plastic deformation is expected in the device since results show a maximum stress of 55 MPa for 350 mmHg. Also, from Fig. 5 we can derive the expected sensitivity of the modeled device to be ~1 μV/mmHg.

## Sensor design, fabrication and packaging

**Fig. 6:**
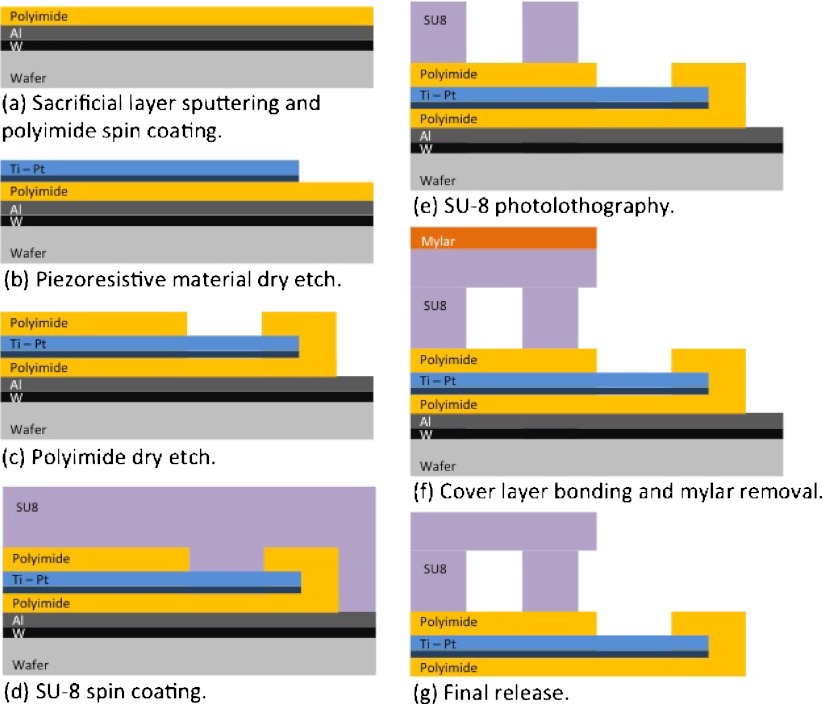
Cross-section view of the fabrication process

The polyimide/SU-8 catheter-tip MEMS gauge pressure sensor contains 2 strain gauges, one active gauge positioned in the middle of the deformable PI membrane and one passive gauge to account for temperature compensation, positioned on the SU-8 wall where no deformation occurs. The resistance of the strain gauges was designed to be 3.2 k*Ω* in order to decrease the power consumption and facilitate readout. The membrane containing the strain gauges was built in polyimide-metal-polyimide sandwich structures by dry etching. The SU-8 pressure chamber was micromachined by standard photolithography and an adapted bonding technique was used to close the chamber with a SU-8 layer. The membrane thickness was selected from the results of the FEA and defined to be 5 μm. The total thickness of the complete device is approximately 105 μm. A cross-sectional view of the microfabrication process is presented in Fig. 6.

**Fig. 7:**
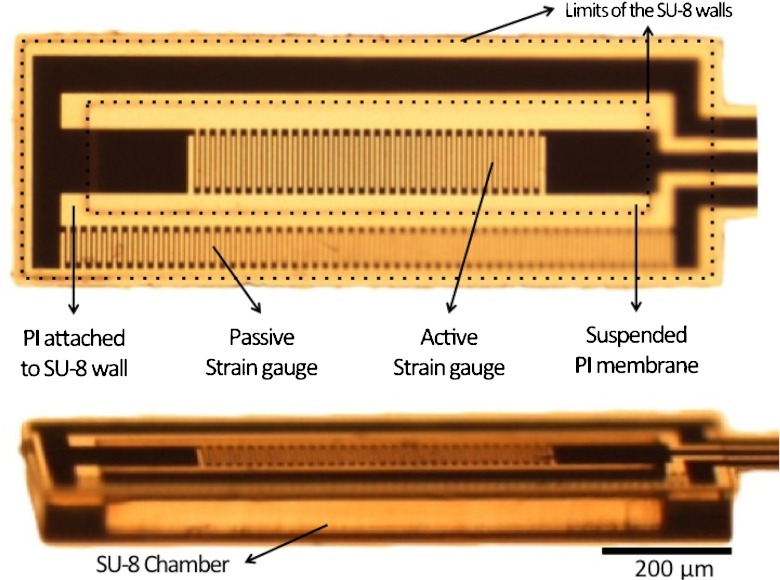
Optical images of a released device comprising the enclosed SU-8 chamber, the active and passive strain gauges, as well as the suspended PI membrane

The detailed microfabrication process comprises the following steps: A sacrificial layer of tungsten (100 nm) and aluminum (1 μm) is first evaporated onto a carrier silicon wafer. A 3 μm layer of PI (PI2611, HD Microsystems) is applied on top of the aluminum by spin-coating and cured at 300 °C for 1 h in nitrogen atmosphere (Fig. 6(a)). A titanium adhesion layer (20 nm) and platinum layer (180 nm) are then sputtered onto the cured polyimide. The strain gauges are patterned by reactive ion etching in Cl_2_ using a patterned photoresist as an etch mask (Fig. 6(b)). A second layer of PI, 2 μm in thickness, is spin-coated and likewise cured. An etch mask of sputtered SiO_2_ (500 nm) is deposited onto the sandwich structure and then patterned by reactive ion etching using a photoresist etch mask. This oxide layer is then used as hard mask during the subsequent oxygen-plasma etch of the polyimide to define both the structure outline and open contact pads to the strain gauges (Fig. 6(c)). A 50 μm layer of SU-8 (GM1070, Gersteltec Engineering Solutions) is applied on top of the polyimide membrane by spin-coating and processed according to the manufacturer specifications (Fig. 6(d)). The SU-8 wall that will support the cover layer is made by photolithography, the development of the SU-8 is performed in propylene glycol methyl ether acetate (PGMEA) after which it is cured at 90 °C for 15 min (Fig. 6(e)). On a second carrier substrate, two thin layers of Ordil^TM^ and one layer of Mylar^TM^ are made to adhere temporarily to the carrier. Afterwards, 50 μm of SU-8 is spun onto the Mylar^TM^ foil. The SU-8 is then pre-baked up to 65 °C, flipped onto the first wafer, contacting the SU-8 sidewalls and then the Mylar^TM^ is removed (Fig. 6(f)). The laminated SU-8 sandwich undergoes photolithography to define the pressure chamber on the underlying device. Development is performed with PGMEA, and laminate is heated up to 90 °C to ensure bonding and cure the epoxy effectively, thus sealing the pressure chamber. The devices are detached from the substrate by anodic metal dissolution in a 10 wt% sodium chloride solution, dissolving the aluminum and releasing the devices (Fig. 6(g)). Optical images of a released device comprising the enclosed SU-8 chamber, the active and passive strain gauges, as well as the suspended PI membrane are shown in Fig. 7. At this stage the device is ready to be packaged into a catheter.

**Fig. 8:**
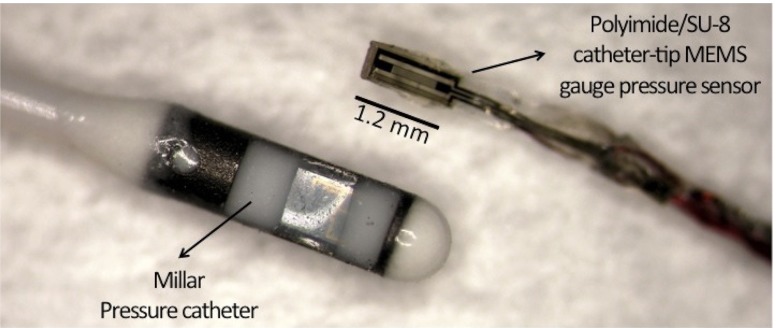
Assembled polyimide/SU-8 catheter-tip MEMS gauge pressure sensor in comparison with a commercial Millar Mikro-Cath^TM^ disposable pressure catheter

The packaging of the device into the Pebax catheter consists of bonding 40 μm diameter insulated copper wires to the device contact pads using a conductive epoxy. This Pebax tube has an inner diameter of 0.20 mm and an outer diameter of 0.48 mm. The copper wires provide the connection between the strain gauges and the complementary electronic circuitry. They were wired through the inner part of the catheter and connected to the rest of the circuit. To protect the connections and attach the device to the tip of the Pebax catheter a standard insulating epoxy was used. The assembled polyimide/SU-8 catheter-tip MEMS gauge pressure sensor is shown in Fig. 8 in comparison with a commercial Millar Mikro-Cath^TM^ disposable pressure catheter fabricated using CMOS technology.

## Experimental setup and procedure

**Fig. 9:**
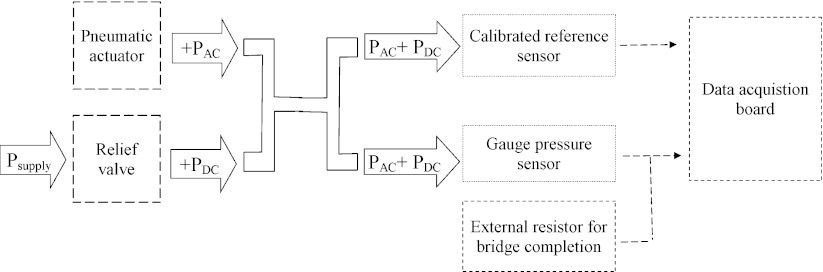
Schematic diagram of the experimental setup

The calibration consists of measuring the voltage output of the device when it is submitted to pressure changes. For this procedure the device and a reference pressure sensor are inserted into a sealed rigid tube. The tube is plugged to a relief valve, which will provide a constant value of pressure (*P*_*DC*_). The relief valve is directly connected to a compressed air supply (*P*_supply_). Pressure variations (*P*_*AC*_) are generated with a pneumatic actuator also connected to the rigid tube. The schematic diagram of the experimental setup is presented in Fig. 9.

The polyimide/SU-8 catheter-tip MEMS gauge pressure sensor is connected in a Wheatstone bridge configuration using two external standard resistors with similar impedance to strain gauges in the membrane. The bridge is powered with 2.5 V and the output signals were recorded with a National Instruments data acquisition board (NI-Daqpad-6015) and a signal conditioning unit (SC-2345) connected to a full-bridge input channel (SCC-SG04). For displaying and recording the measurements a LabView (National Instruments) interface is configured. A calibrated pressure sensor (ENTRAN Pressure Transducer, Model EPX-N01-0.7B) is also attached to the experimental setup and used as reference sensing element for calibrating the device. The reference pressure sensor is powered with 10 V and the signal is acquired using the National Instruments electronic interface previously described, and adding a full-bridge input channel (SCC-SG24). The signal conditioner’s gain and span controls for both sensors are set to obtain a full-scale electrical output signal.

## Experimental Results

**Fig. 10:**
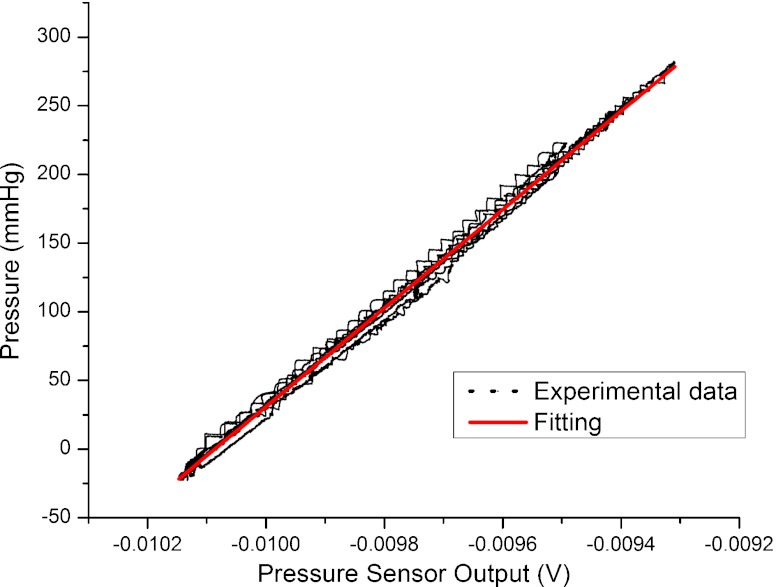
Polyimide/SU-8 gauge pressure sensor calibration *curve* showing reference pressure as a function of the voltage output of the gauge pressure sensor and corresponding linear regression fitting the data

**Fig. 11:**
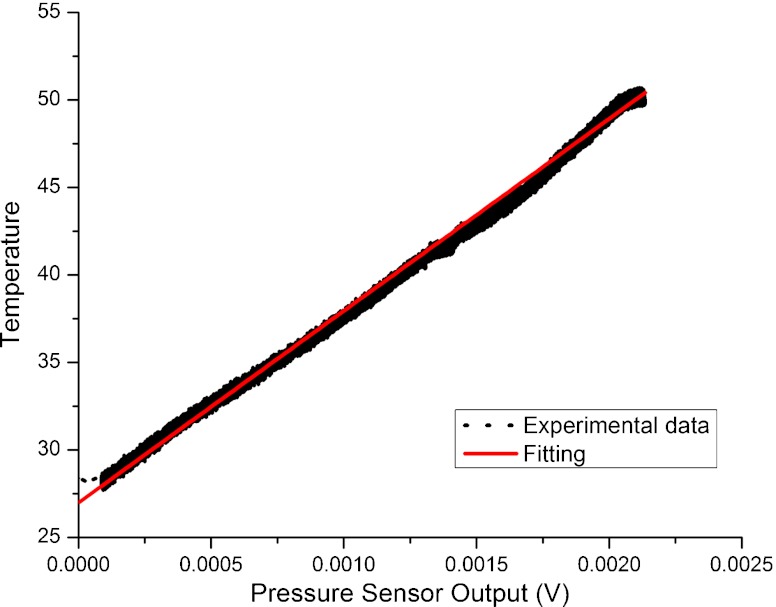
Polyimide/SU-8 gauge pressure sensor response to changes in ambient temperature as a function of the voltage output of the gauge pressure sensor and corresponding linear regression fitting to the data

A time-dependent signal was acquired during the dynamic change in pressure using both sensors, the Polyimide/SU-8 catheter-tip MEMS gauge pressure sensor and the reference pressure sensor. The calibration curve (reference pressure as a function of the voltage output of the gauge pressure sensor) is shown in Fig. 10. The linear regression (adjusted R-square = 0.9982) shows that the gauge pressure sensor has a sensitivity of 2.78 μV/mmHg. The sensor response to changes in temperature is shown in Fig. 11 together with a linear fit of the data (adjusted R-square = 0.9974). This fit implies a temperature sensitivity of 90 μV/°C.

**Fig. 12:**
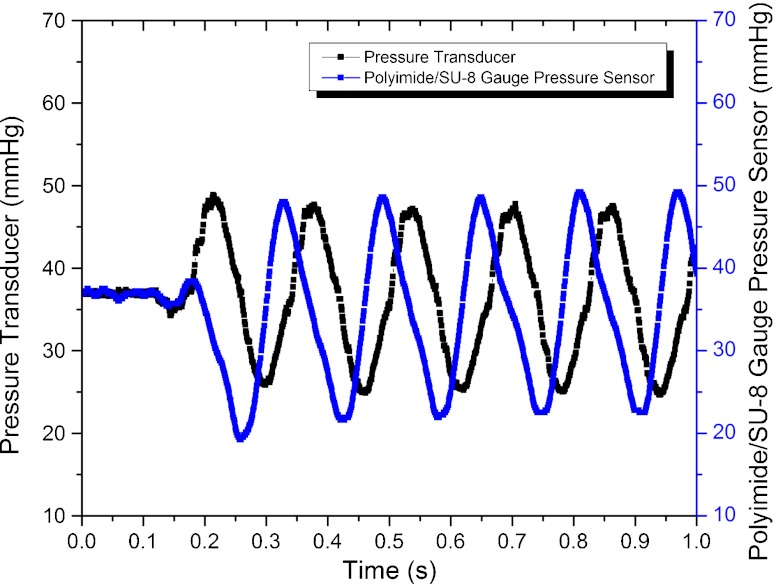
Response of the reference pressure transducer and the polyimide/SU-8 gauge pressure sensor as a function of the time for pressure variations at a frequency of ~7 Hz

Before *in vivo* testing, the performance of the packaged sensor was evaluated in response to an external oscillating pressure. The pressure inside the rigid tube was varied at a frequency of ~7 Hz to emulate the mouse heart beat while the pressure was recorded using both the reference pressure transducer and the polyimide/SU-8 gauge pressure sensors. Results are presented in Fig. 12. The polyimide/SU-8 catheter-tip MEMS gauge pressure sensor performed similarly to the commercial reference sensor. The time-delay observed between the reference sensor and the polymer-based pressure sensor is due to the distance between the sensors in the experimental setup.

## *In vivo* experiment

The goal of the *in vivo* experiment was to prove the device concept and measure mouse blood pressure and heart rate. A male C57BL/6J mouse (weighting 45g) was anesthetized via inhalation of 1–2% isoflurane mixed with oxygen. The left carotid artery was exposed for a length of 5 mm. The fabricated polyimide/SU-8 catheter-tip MEMS gauge pressure sensor was inserted 4 mm into the left carotid artery and tied. The pressure sensor wire was connected to the data-acquisition system to record blood pressure (BP) and heart rate (HR) for about 5 min at a sampling rate of 1 kHz. Anesthesia was maintained by 0.5–1% isoflurane inhalation mixed with oxygen.

**Fig. 13:**
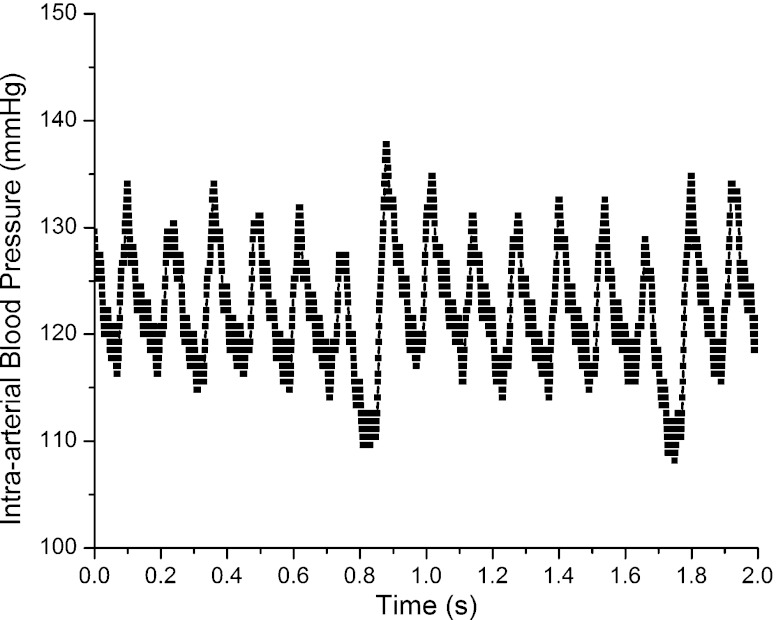
Trace of carotid arterial blood pressure and heart rate in a male C57BL/6J mouse during inhalation of 0.5–1% isoflurane

Figure 13 shows a trace of intra-arterial BP and HR for 2 s. HR is 450 beats/min, systolic BP is 129±1 mmHg and diastolic BP is 115±1 mmHg. The waveform of blood pressure is similar to results obtained with fluid-filled catheters and standard commercial sensors (Wang et al. 2004).

## Discussion

In the present study, a polymer-based catheter-tip gauge pressure sensor was developed for biomedical applications. Experimental results were in good agreement with simulations regarding the device sensitivity. The experimental data demonstrated the sensor has similar characteristics to commercial available devices and can be used in selected biomedical applications (i.e. cardiovascular assessments), and as an alternative to fluid-filled catheters.

The sensitivity of the device can be improved by decreasing the PI membrane thickness and by changing the placement of the strain gauges inside the PI membrane. FEA indicates a nearly 10 fold increase in sensitivity when using a 4 μm thick PI membrane with platinum strain gauges positioned 500 nm from the bottom of the membrane. Optionally, in order to hermetically seal the gauge pressure sensor and improve biocompatibility, a layer of Parylene could be evaporated on the device.

The polyimide/SU-8 catheter-tip MEMS gauge pressure sensor is simple to package and represents a cost effective solution for certain *in vivo* pressure monitoring applications. Adapting the design, improving the packaging and further miniaturizing the pressure sensor will allow not only to insert the device in the carotid artery of mice, but also to place it directly on the heart of the mouse to measure the pressure in the ventricle. Moreover, the device could be integrated with implantable wireless telemetry, which can increase monitoring efficiency, allowing for long time measurements and permitting pressure measurements in awake animals.

## Conclusion

In this work, we demonstrated a polyimide/SU-8 catheter-tip MEMS gauge pressure sensor. Throughout the design process, FEA modeling results were used to optimize the device before fabrication. The polymer-based technology and SU-8 lamination step are well suited for biomedical applications and provide a significant cost advantage over silicon microfabrication techniques. Compared to a silicon-based pressure sensor, the polymer-based device was found to show a similar performance while operating at a lower voltage supply. Finally, the *in vivo* use of this sensor was demonstrated by measuring the heart rate and carotid blood pressure in mice.
